# Awareness, Treatment, and Control of Diabetes in Bangladesh: Evidence from the Bangladesh Demographic and Health Survey 2017/18

**DOI:** 10.1155/2022/8349160

**Published:** 2022-04-22

**Authors:** Nuruzzaman Khan, John C. Oldroyd, Mohammad Bellal Hossain, Rakibul M. Islam

**Affiliations:** ^1^Department of Population Science, Jatiya Kabi Kazi Nazrul Islam University, Mymensingh, Bangladesh; ^2^School of Behavioral and Health Sciences, Australian Catholic University, Fitzroy, Victoria, Australia; ^3^Department of Population Sciences, University of Dhaka, Dhaka, Bangladesh; ^4^School of Public Health and Preventive Medicine, Monash University, Melbourne, Australia; ^5^South Asian Institute for Social Transformation (SAIST), Dhaka, Bangladesh

## Abstract

**Background:**

The prevalence of diabetes is increasing in Bangladesh from ∼5% in 2001 to ∼13% in 2017/18 (∼8.4 million cases). The prevalence of undiagnosed diabetes was also found to be higher at 6% in 2017/18. However, very little is known about the management of diabetes assessed by diabetes awareness, treatment, and control. We aimed to estimate the age-standardised prevalence of awareness, treatment, and control of diabetes and its associated factors.

**Methods:**

Cross-sectional data from 1,174 Bangladeshi adults aged 18 years and older available from the most recent nationally representative Bangladesh Demographic and Health Survey (BDHS) 2017–18 were analysed. Outcomes were age-standardised prevalence of awareness, treatment, and control of diabetes, estimated using the direct standardisation. Multilevel mixed-effects Poisson regression models were used to identify factors associated with awareness, treatment, and control of diabetes.

**Results:**

Of the respondents we analysed, 30.9% (95% CI, 28.2–33.6) were aware that they had the condition, and 28.2% (95% CI, 25.6–30.7) were receiving treatment. Among those treated for diabetes, 26.5% (95% CI, 19.5–33.5) had controlled diabetes. The prevalence of diabetes awareness, treatment, and control was lower in men than women. Factors positively associated with awareness and treatment were increasing age and hypertension, while factors negatively associated with awareness and treatment were being men and lower education. Factors associated with poor control were secondary education and residing in Rajshahi and Rangpur divisions.

**Conclusions:**

This study provides evidence of poor management of diabetes in Bangladesh, especially in men. Less than one-third of the people with diabetes were aware of their condition. Just over one-fourth of the people with diabetes were on treatment, and among those who were treated only one-fourth had controlled diabetes. Interventions targeting younger people, in particular men and those with lower education, are urgently needed. Government policies that address structural factors including the cost of diabetes care and that strengthen diabetes management programmes within primary healthcare in Bangladesh are urgently needed.

## 1. Background

Globally, the prevalence of diabetes mellitus is steadily increasing [1]. Currently, ∼537 million people are living with diabetes worldwide [2]. Without concerted action, this is expected to reach 643 million by 2030 [2]. In addition, 541 million people have impaired glucose tolerance, which puts them at increased risk of developing diabetes [2]. The majority of the global increases in diabetes will occur in low- and lower middle-income countries (LMICs), particularly in Asia and Africa [3]. The rapid increases in diabetes prevalence in LMICs are due to the epidemiological transition resulting in the consumption of westernisation of diets, reduced physical activity, changing patterns in leisure activities, longer working hours, and reduced sleep [4].

Diabetes mellitus was responsible for 6.7 million deaths in 2021 [2]. The majority of these deaths are premature, occurring in younger age groups [5]. Moreover, diabetes management is associated with some 12% of global health expenditure [6]. The Sustainable Development Goals (SDGs) have set a target to reduce premature death from noncommunicable diseases (NCDs) by one-third by 2030 as compared to 2015 levels (SDG Target 3.4) [7]. The effective management of diabetes (awareness, treatment, and control) can reduce diabetes-associated complications, deaths, and costs and can help to achieve the SDGs' target [8, 9].

Bangladesh has one of the highest burdens of diabetes among countries in the Southeast Asian region (∼25 million cases), and there is evidence that this disease burden is increasing [10]. For instance, the prevalence of diabetes in Bangladesh was around 13% in 2017/18 (∼8.4 million cases), increased from only ∼5% in 2001 [10, 11]. The prevalence of undiagnosed diabetes was also found to be higher at 6% in 2017/18 [12]. It is also projected that the current number of cases will be doubled (∼15 million) by 2045 [13]. Although the prevalence of diabetes is increasing in Bangladesh, relevant government healthcare services are limited. There are few government healthcare facilities, mainly district- and division-level hospitals, providing relevant services. These are mainly located in urban areas despite ∼75% of the population living in rural areas [14, 15]. Additionally, the majority of healthcare facilities that provide diabetes management care are private and are for profit [16]. The costs associated with this healthcare effectively reduce access to diabetes services for much of the population [17]. For example, for rural people the associated costs of transport to urban facilities further reduce healthcare access. Given there is no health insurance coverage in Bangladesh, these costs are prohibitive. Therefore, many people cannot go for diabetes diagnosis and/or afford diabetes treatment, which increases diabetes complications and deaths [17].

Several studies have reported a low level of diabetes management in Bangladesh [15, 18–20]. However, these studies are limited by small sample size and/or reported unstandardised estimates. Available studies also only explored diabetes management in selected ages within populations, such as population aged 35 years and more, even though available evidence showed an increasing prevalence of diabetes among population aged <35 years [10, 15]. Moreover, available studies only explored selected sociodemographic characteristics, including age, education, and working status associated with awareness of diabetes [15, 21]. Factors associated with the treatment and control of diabetes have not yet been investigated. To address these limitations, we aimed to examine the age-standardised prevalence of awareness, treatment, and control of diabetes; and factors associated with these conditions among Bangladeshi adults 18 years and older using the latest Bangladesh Demographic and Health Survey (BDHS) 2017–18 data.

## 2. Methods

### 2.1. Data

The data used were extracted from the latest BDHS 2017/18 [21]. This survey is nationally representative and was conducted between 24 October 2017 and 15 March 2018 by the National Institute of Population Research and Training under the Ministry of Health and Family Welfare of Bangladesh and Mitra and Associates. The survey samples were selected using a two-stage stratified random sampling method. In the first stage, 675 primary sampling units (PSUs) were randomly selected from the list of 293 579 PSUs generated as part of the Bangladesh Population and Housing Census 2011. Three PSUs were excluded from the survey due to flood, and data were collected from the remaining 672 PSUs (urban: 192, rural: 480). In the second stage, 30 households were selected from each PSU through probability proportional to the size. This process produced a list of the 20,160 eligible households, and interviews were completed in 19,457 households (overall household response rate of 96.5%) [21]. The survey also collected NCD data, including diabetes and hypertension and their management, at the time of the main survey conducted. For this, one in every four included households in each PSU (7 to 8 households per PSU) was selected. This generated 4,864 households. There were 14,704 eligible respondents aged 18 years or more in these selected households (8,013 women and 6,691 men). Of these, 12,100 respondents had their fasting blood glucose (FBG) measured (6,919 women and 5,181 men). However, diabetes management data were collected from 1,174 respondents, which we analysed in this study. The detailed sampling procedure has been published elsewhere [21].

### 2.2. Outcomes Measures

Awareness, treatment, and control of diabetes were our outcome variables. These outcome variables were generated for the respondents who had diabetes, which was defined as having elevated FBG (≥7 mmol/L) and/or on blood glucose-lowering medication at the time of the survey [21]. Blood glucose was measured using the HemoCue 201 DM system with plasma conversion used to test a drop of capillary blood obtained from consenting eligible respondents from the middle or ring finger. Before collecting blood samples, the participants were requested not to eat or drink except plain water for at least 8 hours before testing. The details on blood sample collection have been described in the BDHS survey report. Awareness of diabetes refers to participants reporting knowing their glucose level as measured before and/or ever been told by a doctor or nurse that they have diabetes. (“*Have you ever been told by a doctor or other health worker that you have high blood sugar or diabetes?*”). Treatment of diabetes refers to participants using medication at the time of the survey to control their diabetes. To collect these data, participants were asked “*Are you currently receiving treatment advice by a doctor or other health worker for your high blood glucose or diabetes?*” Control of diabetes refers to participants using medication to control blood glucose at the time of the survey with the FBG value of less than 7.0 mmol/L [21].

### 2.3. Explanatory Variables

A comprehensive literature review of factors associated with awareness, treatment, and control of diabetes in Bangladesh and its neighbouring countries allowed the identification of the risk factors examined in this study [10, 15, 22, 23]. Factors included were age group (18–34, 35–39, 40–44, 45–49, 50–54, 55–59, 60–64, and ≥65 years), sex (men and women), education (no education/preprimary, primary, secondary, and higher), current working status (yes/no), hypertension (yes or no), wealth quintile (poorest, poorer, middle, richer, and richest), place of residence (urban/rural), and administrative division at the time of the survey. Respondents were considered hypertensive if they had (i) systolic blood pressure ≥140 mmHg and/or a diastolic blood pressure ≥90 mmHg, or (ii) taking any prescribed drugs to control blood pressure [21]. The wealth quintile was generated using principal component analysis based on the respondents' durable and nondurable household goods, such as ownership of land, housing materials, electricity, and television.

### 2.4. Statistical Analysis

We used descriptive statistics to describe the individual, household, and community-level characteristics of the respondents. Age-standardised prevalence estimates of awareness, treatment, and control of diabetes were calculated and presented with 95% CI. The age-standardised estimates were calculated by direct standardisation based on the Bangladesh Population and Housing Census 2011.

We used multilevel mixed-effects Poisson regression with a robust variance to identify factors associated with awareness, treatment, and control of diabetes mellitus. The results were presented as adjusted prevalence ratio (aPR) with 95% CI. Poisson regression was used to avoid overestimation of the odds ratios that occur using logistic regression in cross-sectional studies when the outcome of interest is common [24]. Furthermore, in the BDHS, individuals were nested within the household; households were nested within the PSU/cluster. Therefore, our multilevel mixed-effects Poisson regression model accounts for these multiple hierarchies and dependency in data and the problem of overestimation. Tests for multicollinearity and interaction between explanatory variables were checked before entering into models. All statistical tests were two-sided, and a *p*-value <0·05 was considered statistically significant. The Strengthening the Reporting of Observational Studies in Epidemiology (STROBE) guidelines informed the design and reporting of the study [25]. All analyses were conducted using the statistical software package Stata (version 15·1; Stata Corp LP, College Station, Texas).

## 3. Results

The individual, household, and community-level characteristics of 1,174 participants who provided complete data are summarised in Table 1. The mean (standard deviation) age of the study participants was 46.67 (15.62) years, and 54% were women. A quarter of the participants had no formal education, 46% were employed, and 65% resided in urban areas. Twenty per cent of participants were overweight/obese, and 46% were hypertensive.

The age-standardised prevalence of awareness, treatment, and control of diabetes is presented in Table 2. The overall age-standardised prevalence of awareness was 30.9% (95% CI, 28.2–33.6). People aged ≥65 years had the highest prevalence of awareness (57.0%, 95% CI, 49.5–64.4) compared to other ages. The overall prevalence of awareness in men was 24.5% (95% CI, 21.1–28.0) compared to women 35.0% (95% CI, 31.3–38.7) (*p* < 0.05). The overall prevalence of treatment was 28.2% (95% CI, 25.6–30.7). Treatment was higher among women (32.1%, 95% CI, 28.5–35.7) than men (22.1%, 95% CI, 18.8–25.3) (*p* < 0.05). The prevalence of treatment increased with increasing age, in men and women. The prevalence of control of diabetes was 26.5% (95% CI, 19.5–33.5), increasing with increasing respondent age.

Factors associated with awareness, treatment, and control of diabetes are presented in Table 3. A gradual increase in awareness with increasing respondents' age was observed. Women were more likely to be aware of diabetes than men (aPR, 1.34, 95% CI, 1.08–1.65). Underweight respondents were less likely than normal-weight respondents to be aware of diabetes (aPR, 0.61, 95% CI, 0.40–0.93). Respondents with primary, preprimary, or no education were less likely to be aware (aPR, 0.74, 95% CI, 0.59–0.93) than respondents with higher education. The respondents who belonged to the higher wealth quintile were more likely to be aware (aPR, 1.41, 95% CI, 1.00–1.99) than the lower wealth quintile. Those with hypertension were more likely to be aware (aPR 1.39 (95% CI 1.17–1.64)) than those without hypertension. Awareness of diabetes was less likely among respondents residing in the Dhaka division (aPR, 0.70, 95% CI 0.52–0.95) than those residing in the Barishal division.

We found the likelihood of treatment of diabetes increased with the increase in respondents' age. The highest likelihood of treatment was in respondents aged 60–64 years (aPR 3.88, 95% CI, 2.62–3.75) compared to the respondents aged 18–34 years. Women were more likely to be treated for diabetes than men (aPR, 1.32, 95% CI, 1.06–1.64). Respondents with primary, preprimary, or no education were less likely to be treated than the respondents with higher education (aPR 0.69, 95% CI, 0.54–0.89). Respondents with hypertension were more likely to be treated for diabetes than the respondents without hypertension (aPR 1.44 95% CI, 1.19–1.74).

The likelihood of control of diabetes was found to be 51% (aPR, 0.49, 95% CI, 0.28–0.83) lower among secondary-educated respondents than the higher-educated respondents. We found a lower likelihood of control of diabetes among respondents in Rajshahi (aPR 0.48, 95% CI, 0.27–0.86) and Rangpur (aPR 0.53, 95% CI, 0.29–0.98) division as compared to residents in the Barishal division.

## 4. Discussion

In this nationally representative study of Bangladesh using data from the most recent BDHS 2017–18, we assessed diabetes management by estimating the age-standardised prevalence of awareness, treatment, and control of diabetes and identified factors associated with these conditions. Our findings show that diabetes is poorly managed in Bangladesh, especially in men. Among those with diabetes, only 31% were aware of their condition, and 28% were receiving treatment. Only 26% had controlled diabetes mellitus among those who received treatment. Factors independently associated with awareness and treatment were age, sex, hypertension status, and level of education. Factors associated with poor control were secondary education and residing in Rajshahi and Rangpur divisions.

We found that the prevalence of awareness, treatment, and control of diabetes was low. Only 31% of the total respondents with diabetes knew their diabetes status. Of these, only 28% used medication to control diabetes. Of those using medication to control diabetes, only 26% had controlled diabetes. These indicate poorer management of diabetes in Bangladesh as compared to other Southeast Asian countries. For instance, awareness of diabetes in Bangladesh was lower than the average of Southeast Asian countries (50%) [2], Nepal (65%) [9], and China (49%) [26]. Treatment of diabetes in Bangladesh was also far lower than in Nepal (94%) [9] and China (43%) [26]. However, the control of diabetes in Bangladesh is higher than in Nepal (21%) [9] and China (21%) [26]. The poor management of diabetes in Bangladesh can in part be attributed to the limited provision of and inequitable distribution of government healthcare facilities for diabetes management (e.g. facilities are mainly located in urban areas) [27]. Moreover, misconceptions related to diabetes, including that diabetes only occurs in older people, resulting in lower case detection and treatment among younger populations. Addressing healthcare access and the misconceptions relating to diabetes will be key to improving diabetes management in Bangladesh.

This study found awareness and treatment increased with increasing age, which is comparable with other studies conducted in LMICs, including Bangladesh, India, Nepal, and China [9, 15, 28, 29]. A possible explanation is that as people age, they are more likely to have diabetes, be aware they have it, and have the financial resources to obtain appropriate treatment for it. However, recent data indicate that diabetes prevalence is also increasing in younger people [30], particularly among educated youth [31]. This change in Bangladesh is consistent with an “Asian phenotype” of diabetes, characterised by an onset of diabetes at a younger age and higher risk even at a lower body mass index [32]. Our data suggest that being young does not equate with good diabetes management as awareness and treatment were low in younger people.

We found that people with a low level of education were less likely to be aware, be treated, and have controlled diabetes than those with a higher level of education. Possible mechanisms may be through health literacy and employment. In the social structure of Bangladesh, education is the most important marker of a person's employment. People with lower levels of education are most likely to have manual employment involving physical work. This means that there was worse management of diabetes by a measure of low socioeconomic status, low education, and, by association, manual employment. However, employment appeared to be protective, as there were (nonsignificant) trends to higher awareness, treatment, and control in those with “current employment.” Further investigations of the type of employment (manual vs nonmanual) in relation to diabetes management in Bangladesh are needed.

Though the place of residence was not a significant factor for awareness and treatment of diabetes, we found poorer control of diabetes in respondents of Rajshahi and Rangpur divisions than in Barishal division. Many factors might contribute to such differences in the control of diabetes in these divisions. For instance, Rajshahi and Rangpur divisions are mostly rural and people are mostly engaged in agricultural activity [33]. Consistent with our study, other studies have also found lower awareness, treatment, and control of diabetes among people in these divisions [8] and reported their low level of education and lack of (or difficulties in) access to healthcare facilities [8]

Sex differences in diabetes management were found in this study in which women were more likely to be aware of diabetes and more likely to be treated for diabetes than men. Although not statistically significant, control was also higher in women than men. This is similar to previous findings in Bangladesh [15] and China [29]. The better diabetes management in women could be explained by the higher exposure of women to maternal and child health services provided by the community healthcare workers at the household level. As such, women are more likely to discuss a range of health issues, including gestational diabetes, with health workers compared with men [34, 35]. Efforts, particularly in men, are needed to ensure good diabetes management [36].

Our study shows that people being underweight were less aware of their diabetes condition compared with people having normal weight. One plausible explanation could be the strong association of diabetes with obesity in health promotion literature, reducing the awareness of underweight people about their risk.

The National Guideline for Diabetes Management in Bangladesh focused on raising diabetes awareness and associated factors [37]. These included unhealthy diets, physical activity, and overweight or obesity [37] because of the strength of the evidence of their protective effects [38–40]. However, the roll-out is currently limited. For instance, public health measures and programmes to increase awareness and treatment of diabetes are still restricted to urban centres and are small in scale, mostly organised by the Diabetic Association of Bangladesh [41]. Also, public health campaigns alone have not proven effective in preventing diabetes though they can increase awareness [42]. Other innovative approaches including the use of social media and mobile phone text messaging could be cost-effective interventions to improve glycaemic control in patients with diabetes [43]. This is particularly the case as the government of Bangladesh has adopted information technologies for health in its strategic plans [43].

The implications of our findings are that there needs to be substantial investment in health promotion to raise awareness and changes in healthcare delivery that address treatment and control of diabetes regardless of sociodemographic status. However, the management of diabetes within the healthcare sector in Bangladesh is challenging for several reasons. The major challenge comes from the facility level, in which maternal and child health and other diseases are prioritised [27]. So far in Bangladesh, the establishment of NCDs' corners at the Upazila Health Complexe is the only programme that the government of Bangladesh has taken to reduce diabetes and other NCDs [44]. People with diabetes, therefore, mostly depend on private healthcare facilities and services provided by the Bangladesh Diabetes Federation, a nonprofit specialised organisation for managing diabetes. However, their services are mostly located in urban areas, and private healthcare facilities are expensive [27]. Therefore, the management of diabetes is challenging for poor and rural people due to structural and economic difficulties given that there is no healthcare insurance coverage in Bangladesh.

A limitation of this study was that outcome data are self-reported. Diet, metabolic, lifestyle, and behavioural factors are important determinants for diabetes management. However, these data were not available in the dataset and so could not be considered in our analyses. Moreover, the design of this survey was cross-sectional, which limits our capacity to draw casual associations. The major strength is that this is the first study in Bangladesh using a large nationally representative dataset that included adults 18 years and older, suggesting the findings have external validity. Our study generates findings with increased precision because of the use of multilevel mixed-effects Poisson regression that corrects the overestimation of effect size produced by conventional logistic regression employed in cross-sectional studies.

## 5. Conclusions

This study provides evidence of poor management of diabetes in Bangladesh, especially in men. Less than one-third of the people with diabetes were aware of their condition. Just over one-fourth of the people with diabetes were on treatment, and among those who were treated only one-fourth had controlled diabetes. Interventions targeting younger people, in particular men and those with lower education, are urgently needed. Government policies that address structural factors including the costs of diabetes care and that strengthen diabetes management programmes within primary healthcare in Bangladesh are urgently needed.

## Figures and Tables

**Table 1:** Background characteristics of the study population (*N* = 1,174).

Characteristics	Number (%)
Age (years), mean (SD)	46.77 (15.62)
18–34	277 (23.6)
35–39	138 (11.7)
40–44	131 (11.2)
45–49	133 (11.3)
50–54	115 (9.8)
55–59	100 (8.5)
60–64	112 (9.5)
≥65	168 (14.3)
Sex	
Men	537 (45.8)
Women	637 (54.2)
Body mass index, mean (SD)	24.05 (4.46)
Underweight (<18.5)	128 (10.9)
Normal weight (18.5–22.9)	396 (33.7)
Overweight (23.0–27.5)	425 (36.2)
Obese (≥27.5)	225 (19.1)
Education	
No education/preprimary	296 (25.2)
Primary	365 (31.1)
Secondary	337 (28.7)
Higher	176 (15.0)
Currently working	
Yes	636 (54.1)
No	538 (45.9)
Hypertension	
Yes	538 (45.8)
No	636 (54.2)
Wealth quintile	
Poorest	128 (10.9)
Poorer	141 (12.0)
Middle	194 (16.5)
Richer	266 (22.7)
Richest	445 (37.9)
Place of residence	
Rural	415 (35.4)
Urban	758 (64.6)
Region of residence	
Barishal	62 (5.3)
Chattogram	224 (19.1)
Dhaka	394 (33.6)
Khulna	121 (10.3)
Mymensingh	77 (6.5)
Rajshahi	138 (11.7)
Rangpur	84 (7.1)
Sylhet	74 (6.3)

Note: all numbers and percentages are weighted. SD = standard deviation.

**Table 2:** Age-standardised prevalence of awareness, treatment, and control of diabetes by sex, Bangladesh 2017/18.

Age in years	Awareness			Treatment			Control		
	Overall (453/1174)	Men (198/537)	Women (255/637)	Overall (413/1174)	Men (178/537)	Women (235/637)	Overall (126/413)	Men (58/178)	Women (68/235)
18–34	15.1 (10.8–19.3)	6.8 (1.9–11.7)	19.9 (14.0–25.8)	13.3 (9.3–17.3)	5.8 (1.3–10.4)	17.6 (12.0–23.3)	21.6 (8.1–35.1)	16.7 (−16–49.6)	22.6 (7.5–37.6)
35–39	32.9 (25.0–40.8)	28.6 (16.6–40.5)	35.7 (25.4–46.0)	30.0 (22.4–37.6)	25.0 (13.5–36.5)	33.3 (23.2–43.5)	26.2 (12.7–39.7)	28.6 (3.8–53.3)	25.0 (8.6–41.4)
40–44	37.9 (29.3–46.5)	25.0 (13.5–36.5)	48.5 (36.5–60.5)	33.1 (24.7–41.4)	21.4 (10.6–32.3)	42.6 (30.8–54.5)	29.3 (15.1–43.4)	25.0 (−1–50.8)	31.0 (13.8–48.3)
45–49	51.5 (42.9–60.1)	46.7 (31.9–61.4)	54.0 (43.4–64.6)	47.7 (39.2–56.3)	40.0 (25.5–54.5)	51.7 (41.1–62.3)	30.2 (18.7–41.6)	33.3 (10.7–55.9)	28.9 (15.4–42.3)
50–54	48.6 (39.3–58.0)	36.4 (23.5–49.2)	60.7 (47.8–73.6)	45.0 (35.7–54.4)	34.5 (21.8–47.3)	55.4 (42.2–68.5)	30.0 (17.1–42.9)	36.8 (14.4–59.3)	25.8 (10.1–41.5)
55–59	59.1 (49.9–68.3)	64.4 (50.3–78.6)	55.4 (43.2–67.6)	55.4 (46.1–64.8)	64.4 (50.3–78.6)	49.2 (37.0–61.5)	29.5 (17.1–42.9)	27.6 (10.9–44.3)	31.3 (14.8–47.6)
60–64	55.2 (45.7–64.8)	60.3 (47.6–73.1)	48.9 (34.5–63.4)	52.4 (42.8–62.0)	56.9 (44.0–69.8)	46.8 (32.4–61.3)	34.5 (21.8–47.3)	33.3 (16.9–49.8)	36.4 (15.7–57.0)
≥65	57.0 (49.5–64.4)	58.2 (48.3–68.0)	55.4 (44.0–66.8)	52.9 (45.4–60.4)	52.0 (42.1–62.0)	54.1 (42.6–65.5)	41.8 (21.8–47.3)	35.3 (21.9–48.6)	50.0 (34.2–65.8)
Total	30.9 (28.2–33.6)	24.5 (21.1–28.0)	35.0 (31.3–38.7)	28.2 (25.6–30.7)	22.1 (18.8–25.3)	32.1 (28.5–35.7)	26.5 (19.5–33.5)	23.9 (8.5–39.4)	27.4 (19.4–35.4)

Note: diabetes was defined as having elevated fasting blood glucose (FBG, ≥7 mmol/L) and/or on blood glucose-lowering medication at the time of the survey; awareness of diabetes refers that the respondents ever been told by a doctor or nurse that they have diabetes; treatment of diabetes refers to respondents using medication to control their diabetes; and control of diabetes refers to treated diabetes with FBG value less than 7.0 mmol/L.

**Table 3:** Factors associated with the awareness, treatment, and control of diabetes in Bangladesh, 2017/18.

	Awareness-adjusted PR (95% CI)	Treatment-adjusted PR (95% CI)	Control-adjusted PR (95% CI)
Individual level			
Age in years (ref: 18–34)			
35–39	2.12 (1.46–3.07)^*∗∗∗*^	2.30 (1.53–3.44)^*∗∗∗*^	0.95 (0.43–2.12)
40–44	2.45 (1.65–3.64)^*∗∗∗*^	2.49 (1.61–3.84)^*∗∗∗*^	1.37 (0.63–2.96)
45–49	3.11 (2.19–4.43)^*∗∗∗*^	3.21 (2.19–4.71)^*∗∗∗*^	1.93 (0.93–4.03)
50–54	3.10 (2.14–4.48)^*∗∗∗*^	3.22 (2.15–4.82)^*∗∗∗*^	1.21 (0.52–2.79)
55–59	3.44 (2.42–4.91)^*∗∗∗*^	3.69 (2.49–5.45)^*∗∗∗*^	1.23 (0.57–2.65)
60–64	3.65 (2.53–5.27)^*∗∗∗*^	3.88 (2.62–3.75)^*∗∗∗*^	1.75 (0.83–3.69)
≥65	3.70 (2.63–5.21)^*∗∗∗*^	3.72 (2.54–5.45)^*∗∗∗*^	1.92 (0.97–3.81)
*Sex (ref: men)*	1.34 (1.08–1.65)^*∗∗∗*^	1.32 (1.06–1.64)^*∗∗∗*^	1.04 (0.69–1.56)
Body mass index (kg/m^2^) (ref: normal weight)			
Underweight (<18.5)	0.61 (0.40–0.93)^*∗∗*^	0.68 (0.44–1.04)	1.68 (0.99–2.86)
Overweight (23.0–27.5)	0.98 (0.82–1.18)	0.97 (0.80–1.19)	0.79 (0.55–1.14)
Obese (≥27.5)	1.01 (0.81–1.26)	0.99 (0.78–1.26)	1.09 (0.70–1.70)

Education (ref: higher education)			
No education/preprimary/primary	0.74 (0.59–0.93)^*∗∗∗*^	0.69 (0.54–0.89)^*∗∗∗*^	0.81 (0.54–1.23)
Secondary	1.03 (0.83–1.28)	0.97 (0.76–1.23)	0.49 (0.29–0.83)^*∗∗∗*^
*Currently working (ref: no)*	*1.07 (0.89–1.29)*	*1.02 (0.84–1.24)*	*1.31 (0.90–1.92)*
*Hypertension (ref: no)*	1.39 (1.17–1.64)^*∗∗∗*^	1.44 (1.19–1.74)^*∗∗∗*^	0.91 (0.68–1.22)

Household level			
Wealth quintile (ref: lowest)			
Second	0.80 (0.55–1.18)	0.72 (0.48–1.08)	0.68 (0.35–1.31)
Middle	1.11 (0.78–1.58)	0.97 (0.67–1.41)	1.02 (0.64–1.61)
Fourth	1.17 (0.83–1.64)	1.07 (0.75–1.53)	0.63 (0.36–1.11)
Highest	1.41 (1.00–1.99)^*∗∗*^	1.27 (0.87–1.82)	0.65 (0.40–1.06)

Community level			
*Place of residence (ref: rural)*	0.91 (0.77–1.07)	0.94 (0.79–1.11)	1.06 (0.76–1.47)
Administrative division (ref: Barishal)			
Chattogram	1.01 (0.78–1.32)	1.03 (0.77–1.35)	0.75 (0.44–1.27)
Dhaka	0.70 (0.52–0.95)	0.68 (0.50–0.93)	0.60 (0.34–1.04)
Khulna	0.97 (0.73–1.28)	1.00 (0.76–1.34)	0.73 (0.41–1.29)
Mymensingh	0.97 (0.71–1.33)	0.99 (0.71–1.39)	0.95 (0.54–1.70)
Rajshahi	1.19 (0.90–1.58)	1.09 (0.81–1.46)	0.48 (0.27–0.86)^*∗∗∗*^
Rangpur	1.19 (0.88–1.60)	1.28 (0.94–1.73)	0.53 (0.29–0.98)^*∗∗*^
Sylhet	0.86 (0.63–1.16)	0.90 (0.66–1.21)	1.04 (0.61–1.77)

aPR = adjusted prevalence ratio,  ^*∗*^ ^*∗*^ ^*∗*^*p* < 0.01 and  ^*∗*^ ^*∗*^*p* < 0.05.

## Data Availability

The Demography and Health Survey (DHS) programme of the USA is the custodian of 2017 BDHS data. It is freely available for the user upon submission of reasonable request to the DHS. [28]

## Abbreviations

LMICs: Low- and lower middle-income countries
SDGs: Sustainable Development Goals
NCDs: Noncommunicable diseases
BDHS: Bangladesh Demographic and Health Survey
aPR: Adjusted prevalence ratio, 95% CI, 95% confidence interval.

## Additional Points

(i) What is already known? The prevalence of diabetes is rapidly increasing in low- and lower middle-income countries (LMICs). The increase is mostly associated with the increasing rate of overweight and obesity and is concentrated among people of high socioeconomic status. However, evidence of the management of diabetes in LMICs including Bangladesh is scarce. (ii) What does this article add? We report that the management of diabetes in Bangladesh is poor. Only 31% of the total respondents with diabetes were aware that they had the condition; of them, only 28% were taking treatment. Among those treated for diabetes, only 26% had controlled diabetes. Increasing age and hypertension status were found to be associated with greater awareness and treatment of diabetes. The factors negatively associated with awareness and treatment of diabetes were being men and having lower education.

## Supplementary Materials

Supplementary Materials STROBE statement—Checklist of items that should be included in reports of *cross-sectional studies*.
